# Effect of Short-Term Increase in Meal Frequency on Glucose Metabolism in Individuals with Normal Glucose Tolerance or Impaired Fasting Glucose: A Randomized Crossover Clinical Trial

**DOI:** 10.3390/nu11092126

**Published:** 2019-09-06

**Authors:** Masanobu Hibi, Sayaka Hari, Tohru Yamaguchi, Yuki Mitsui, Sumio Kondo, Mitsuhiro Katashima

**Affiliations:** 1 Biological Science Research Laboratories, Kao Corporation, 2-1-3 Bunka, Sumida-ku, Tokyo 131-8501, Japan; 2 Personal Health Care Laboratories, Kao Corporation, 2-1-3, Bunka, Sumida-ku, Tokyo 131-8501, Japan; 3 Health Care Food Research Laboratories, Kao Corporation, 2-1-3 Bunka, Sumida-ku, Tokyo 131-8501, Japan; 4 Fukushima Healthcare Center, Kensyokai Medical Corporation, 2-12-16, Tamakawa, Fukushima-ku, Osaka 553-0004, Japan

**Keywords:** meal frequency, glucose, CGMS, GLP-1, insulin

## Abstract

Effects of meal frequency on blood glucose levels and glucose metabolism were evaluated over 3 days in adult males with normal glucose tolerance (NGT, *n* = 9) or impaired fasting glucose (IFG, *n* = 9) in a randomized, crossover comparison study. Subjects were provided with an isocaloric diet 3 times daily (3M) or 9 times daily (9M). Blood glucose was monitored on Day 3 using a continuous glucose monitoring system, and subjects underwent a 75-g oral glucose tolerance test (OGTT) on Day 4. Daytime maximum blood glucose, glucose range, duration of glucose ≥180 mg/dL, and nighttime maximum glucose were significantly lower in the NGT/9M condition than in the NGT/3M condition. Similar findings were observed in the IFG subjects, with a lower daytime and nighttime maximum glucose and glucose range, and a significantly higher daytime minimum glucose in the 9M condition than in the 3M condition. The OGTT results did not differ significantly between NGT/3M and NGT/9M conditions. In contrast, the incremental area under the curve tended to be lower and the maximum plasma glucose concentration was significantly lower in the IFG/9M condition than in the IFG/3M condition. In IFG subjects, the 9M condition significantly improved glucose metabolism compared with the 3M condition. Higher meal frequency may increase glucagon-like peptide 1 secretion and improve insulin secretion.

## 1. Introduction

Type 2 diabetes mellitus (T2DM), and the stage preceding T2DM, i.e., impaired glucose tolerance, are major risk factors for cardiovascular disease [1]. A prolonged hyperglycemic state due to poor blood glucose control can lead to complications such as neurologic disorders, retinopathy, and nephropathy [2,3]. Large-scale studies indicate an association between postprandial glucose level and both cardiovascular disease and all-cause mortality [4,5]. Thus, for patients with impaired glucose tolerance or T2DM, suppressing the rapid increase in postprandial glucose (PPG) may be more important than achieving target hemoglobin A1c (HbA1c) levels and fasting blood glucose levels [6,7,8,9,10,11]. The International Diabetes Federation published the following guidelines: fasting blood glucose ≤100 mg/dL (≤5.5 mmol/L), HbA1c <6.5%, and 2-h PPG ≤140 mg/dL (≤7.8 mmol/L) [12]. The American Diabetic Association’s 2010 Standards of Medical Care in Diabetes recommends blood glucose control that maintains peak PPG levels at <180 mg/dL (≤10 mmol/L) [13]. Moreover, intermittent high glucose may be more harmful than stable high glucose, as suggested by cell-based studies showing that acute increases in blood glucose or large fluctuations in glucose increase oxidative stress, protein kinase C activation [14], apoptosis [15], and inflammation [16].

Daily meal frequency is influenced not only by biologic factors (e.g., hunger) and habit, but also by social factors such as lifestyle and/or occupation, and is thought to influence weight change and glucose tolerance. Increasing meal frequency under isocaloric conditions affects fluctuations in PPG and insulin. Studies of T2DM patients by Jenkins et al. [17] and Bertelsten et al. [18] revealed that maintaining a constant total amount of food intake while increasing the meal frequency leads to a reduction in peak PPG. Increasing meal frequency under isocaloric conditions in healthy individuals improves fat metabolism, although improved glucose metabolism has not been demonstrated conclusively [19,20]. In these studies, PPG was measured intermittently and thus the effects of the interventions on continuous fluctuations in blood glucose throughout the day and on glucose metabolism remain unclear. Continuous glucose monitoring systems (CGMS) allow for continuous glucose measurements and are useful for assessing the effects of interventions on appetite and glucose concentrations throughout the day. Ohkawara et al. [21] and Munsters and Saris [22] assessed the relationship between meal frequency and 24-h glucose fluctuations in healthy adults and reported different results. Furthermore, the effect of increasing meal frequency on glucose metabolism and the effect of glucose tolerance on the relationship between meal frequency and improvements in peak PPG are both unknown.

We evaluated the effect of meal frequency over the course of a 3-day intervention period on daily fluctuations in blood glucose and subsequent glucose metabolism in adult male subjects with normal glucose tolerance (NGT) or impaired fasting glucose (IFG). Fluctuations in 24-h glucose during the intervention were tracked using a CGMS, and glucose metabolism was evaluated by an oral glucose tolerance test (OGTT).

## 2. Materials and Methods

### 2.1. Subjects

Healthy men with NGT or IFG were recruited via websites and posters. Inclusion criteria were as follows: (1) age between 30 and 60 years, (2) fasting blood glucose ≤100 mg/dL and HbA1c ≤5.8% for men with NGT, and fasting blood glucose 110–126 mg/dL and HbA1c 5.8–6.4% for men with IFG. Exclusion criteria were as follows: (1) diabetes patients and/or individuals with hepatic dysfunction, kidney disease, or any other serious disease, (2) smokers, (3) heavy alcohol users (≥30 g/day), (4) shift-workers and night-workers, and (5) those considered unsuitable to participate in the study by the study physician. The sample size, based on the results of a preliminary study, was set at 10 per group to achieve a significance level of 5% with a detection power of 0.8. DRC Co., Ltd. (Osaka, Japan) was commissioned to carry out all tasks related to the trial, including subject recruitment and randomization. The trial was conducted from January 2010 to March 2011. This trial was approved by the ethics review committee of Fukushima Healthcare Center, Kenshokai Medical Corporation (Osaka, Japan, Approval date: 29 December 2010), conducted in accordance with the ethical principles set forth in the Declaration of Helsinki, and complied with the study protocol. Details of the trial were sufficiently explained to subjects both verbally and in writing, and written informed consent was obtained from all subjects. This trial was registered with the University Hospital Medical Information Network (UMIN; http://www.umin.ac.jp/; Registration No. UMIN000036661).

### 2.2. Study Design

**Figure 1:**
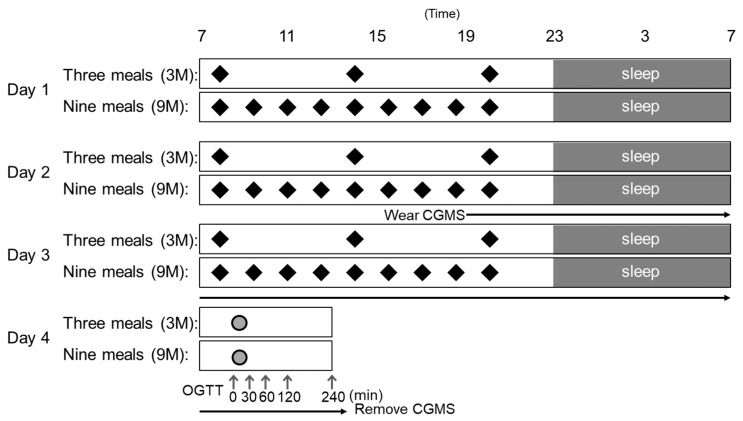
Schematic of the study design. During the trial period, subjects were allocated to receive 9 meals/day (9M) or 3 meals/day (3M) over the course of 3 days, with an oral glucose tolerance test (OGTT) conducted after the last day of the intervention period. The interventions were switched after at least a 2-week washout. In the 3M condition, the subjects were provided meals at 08:00, 14:00, and 20:00 on Days 1-3 of the intervention period. In the 9M condition, the same meals were divided into 9 meals, which were provided at 08:00, 09:30, 11:00, 12:30, 14:00, 15:30, 17:00, 18:30, and 20:00. The continuous glucose monitoring systems (CGMS) was worn by subjects starting at 18:00 on Day 2 up to 13:00 on Day 4. Approximately once every 6 h, the subjects performed calibrations using a self-monitoring blood glucose device. Subjects showered between 20:00 and 23:00 on Days 1 and 2, slept at 23:00, and woke up the next morning at 07:00. From Day 1 to Day 3, subjects drank water freely. On Day 4, subjects remained in a fasted state (no water, no food) after waking up, and the 75-g OGTT was performed. Gray area: sleep; black diamonds: meals; gray circles: intake of 75-g glucose solution; gray arrows: blood collection.

This study had a randomized, cross-over design. The intervention involved providing subjects with a 3-meal per day diet (3M) or a 9-meal per day diet (9M) over the course of 3 days, with the conditions switched after a minimum wash-out period of 2 weeks. A schematic summarizing the study design is provided in Figure 1. The subjects were allocated through stratified randomization with the stratification factors of body mass index (BMI) and age. During the intervention period (Day 1 to Day 3), subjects were instructed to eat only those meals provided by the study coordinator. The subjects were also asked to avoid strenuous exercise and intake of alcohol and caffeine the day before the intervention period. On Days 1 and 2, the subjects arrived at the hospital at 08:30, and stayed in the outpatient unit (Shoji Clinic, Osaka, Japan) until they left at 21:30. At 20:00 on Day 2, a CGMS was placed in the subcutaneous tissue of the abdomen to continuously monitor blood glucose until the end of each intervention period (Day 4). On Day 3, subjects arrived at the hospital at 08:30, went to sleep at 23:00, woke up the next day (Day 4) at 07:00, and left the outpatient unit at 13:00. After measuring body weight, body fat percentage, blood pressure, and body temperature, a 75-g OGTT (TRELAN-G75; Ajinomoto Co., Inc., Tokyo, Japan) was performed starting at 09:00 on Day 4.

### 2.3. Test Meals

During the 3-day intervention period, all meals were provided by the study coordinator. Meals for the 3M and 9M conditions were prepared with commercial foods such that the amount provided corresponded to the basal metabolic rate calculated from the age, weight, and height of each subject [23] multiplied by an activity factor of 1.6 (i.e., 30% of energy from fat, 55% from carbohydrate, and 15% from protein). All meals were designed by a registered dietitian based on nutritional value calculations using Healthy Maker Pro software (Mushroom Soft Co., Ltd., Okayama, Japan) in accordance with the Standard Tables for Food Composition in Japan. In the 3M condition, meals were provided at 6-h intervals at 08:00, 14:00, and 20:00. In the 9M condition, meals were provided at 1.5-h intervals at 08:00, 09:30, 11:00, 12:30, 14:00, 15:30, 17:00, 18:30, and 20:00 (Figure 1). In terms of energy distribution, each meal was adjusted to correspond to 33.3% of the daily recommended caloric intake for the 3M condition, and 11.1% for the 9M condition. Because the trial was conducted under the supervision of the study coordinator, test meal intake rates for both conditions were 100%.

### 2.4. Measurements

Subjects were instructed to avoid consuming food and drink other than water from 21:00 the day before blood collection up until fasting blood collection the next morning. Morning fasting blood was collected from a peripheral vein and used to measure total cholesterol, high-density lipoprotein (HDL)-cholesterol, low-density lipoprotein (LDL)-cholesterol, triglycerides, free fatty acids, insulin, fasting blood glucose, and HbA1c. A CGMS (Medtronic MiniMed, Northridge, CA, USA) was used to measure blood glucose during the intervention period in 5-min increments starting on the night of Day 2 until the morning of Day 4 (i.e., the day after the 3-day intervention period) [24]. A physician attached the CGMS to the subcutaneous tissue of the abdomen at 20:00 on Day 2. After that, the subjects calibrated the CGMS every 4-6 h using a self-monitoring blood glucose device (Accu-Chek^®^; Roche Diagnostics) and inputted the data into the CGMS [25]. After the OGTT on Day 4, the CGMS was removed at 13:30 and data were collected. Blood glucose data from 07:00 on Day 3 to 07:00 on Day 4 were used to calculate mean blood glucose, area under the curve (AUC), maximum glucose, minimum glucose, glucose range, duration of blood glucose ≥180 mg/dL, and duration of blood glucose ≤70 mg/dL.

A 75-g OGTT was performed on Day 4. Food and drink other than water were prohibited from 21:00 the day before blood collection up until fasting blood collection the next morning. Fasting blood was collected from a peripheral vein starting at 09:00, and 30 min (09:30), 1 h (10:00), 2 h (11:00), and 4 h (13:00) after drinking the 75-g glucose solution. Blood samples for measurements of HbA1c, insulin, glucagon, total glucose-dependent insulinotropic polypeptide (GIP), and active glucagon-like peptide (GLP)-1 were collected into tubes containing EDTA-2Na, and blood samples for measuring glucose were collected into tubes containing NaF-EDTA. The tubes were centrifuged at 2000g for 15 min at 4 °C to obtain plasma samples. The other blood biochemistries were measured using serum samples obtained by centrifugation (4 °C, 2000 g, 15 min) of the blood samples after standing at room temperature for 15 min. Total cholesterol, HDL-cholesterol, LDL-cholesterol, triglyceride, free fatty acids, insulin, fasting blood glucose, ketone bodies, glucagon, and HbA1c were measured by SRL (Tokyo, Japan). Total GIP and active GLP-1 were measured by enzyme-linked immunosorbent assay (LINCO Research, MO, USA). Fasting levels, incremental AUC (iAUC), and maximum blood level (Cmax) of glucose, insulin, glucagon, total GIP, and active GLP-1 were calculated from values obtained at the time of measurement. The homeostatic model assessment of insulin resistance (HOMA-IR), QUICKI, Matsuda index, and insulinogenic index were calculated from the glucose and insulin values obtained during the OGTT [26,27,28,29].

### 2.5. Statistical Analysis

All data are presented as mean ± standard deviation. Analyses were performed using data from all subjects who completed the trial (full analysis set). Baseline values for subjects with NGT or IFG were assessed using Student’s *t*-test. Measured values during and after the intervention were evaluated for equality of variance using the paired *t*-test. Fluctuations in glucose, insulin, and gastrointestinal hormones were assessed by analysis of variance using a mixed model. The level of significance was set at 5%, and *p* values less than 0.05 were considered statistically significant. Statistical analyses were performed using the SAS system for Windows (Ver. 9.2, SAS Institute, Cary, NC).

## 3. Results

### 3.1. Subjects

Nineteen subjects were recruited through websites and posters. Blood glucose could not be assessed in one subject due to a malfunctioning CGMS. Accordingly, data from 18 subjects (9 subjects with NGT and 9 subjects with IFG; mean age ± SD, 49 ± 7 years; mean BMI ± SD, 25.9 ± 4.0 kg/m^2^) who completed the trial were analyzed. Baseline physical data and blood-related parameters are provided in Table 1. Compared to subjects with NGT, those with IFG had significantly higher HbA1c, fasting blood glucose, and HOMA-IR. The physical parameters and blood pressure did not differ significantly between subjects with NGT and those with IFG. Because the test food compliance rate was 100% during the intervention period, meal frequency had no effect on average daily total energy intake, protein intake, fat intake, or glucose intake.

### 3.2. Continuous Glucose Monitoring Systems

**Figure 2:**
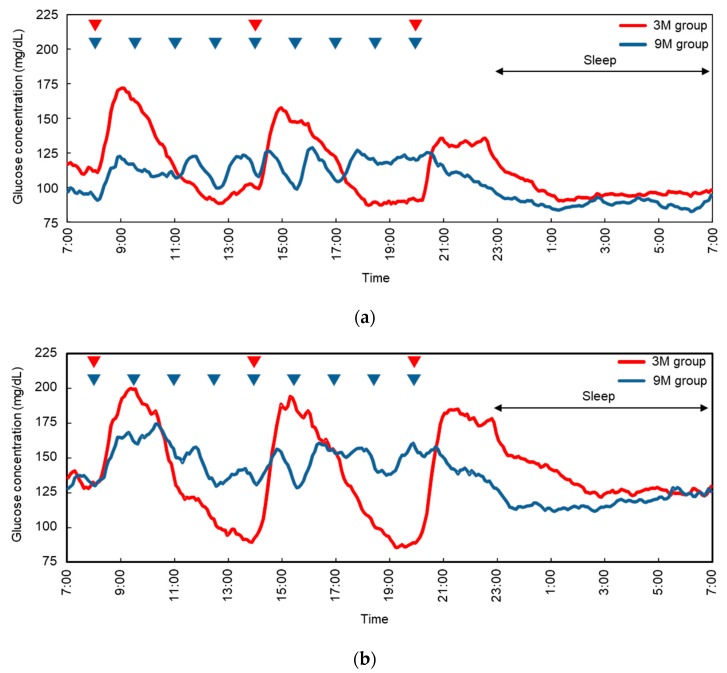
24-h glucose concentrations as measured by CGMS in (**a**) subjects with normal glucose tolerance (NGT, *n* = 9) and (**b**) subjects with impaired fasting glucose (IFG, *n* = 9). Red line: 3-meal (3M) condition; blue line, 9-meal (9M) condition. In the 3M condition, the meals were provided at 08:00, 14:00, and 20:00. In the 9M condition, the same meals were divided into 9 meals provided at 08:00, 09:30, 11:00, 12:30, 14:00, 15:30, 17:00, 18:30, and 20:00.

Blood glucose during the last 24 h of the 3-day intervention period was monitored with a CGMS (Figure 2). The 24-h glucose fluctuation patterns clearly differed between the 3M and 9M conditions in both the NGT and IFG groups. The 24-h glucose profiles measured by the CGMS are provided in Table 2. Mean 24-h blood glucose did not differ significantly between the 3M and 9M conditions for either the NGT or IFG groups. In the 9M condition, NGT subjects had significant reductions in daytime (07:00–23:00) maximum blood glucose, glucose range, duration of glucose ≥180 mg/dL, and nighttime (23:00–07:00) maximum blood glucose, compared with the 3M condition. Similar trends were observed in the IFG subjects, with reductions in daytime and nighttime maximum blood glucose and glucose range in the 9M condition compared with the 3M condition. Subjects with IFG also exhibited significantly increased daytime minimum glucose concentrations in the 9M condition compared with the 3M condition.

### 3.3. Glucose Metabolism

Aggregate iAUC and Cmax values of blood glucose, insulin, glucagon, GLP-1, GIP as assessed by the OGTT after the 3-day intervention period are provided in Table 3. In the NGT group, fluctuations in blood glucose, insulin, glucagon, GLP-1, and GIP up to the end of the OGTT (240 min after drinking the 75-g glucose solution) did not differ significantly between the 9M and the 3M conditions. Analysis of temporal data using a linear mixed effects model revealed the lack of a main effect of the intervention on blood glucose and insulin fluctuations (*p* = 0.398 and *p* = 0.269, respectively), the lack of an effect of time (*p* = 0.938 and *p* = 0.263, respectively), and the lack of an intervention × time interaction (*p* = 0.896 and *p* = 0.125, respectively).

In contrast, in the IFG group, the Cmax of blood glucose was significantly reduced in the 9M condition compared with the 3M condition, and there was a trend toward reduced iAUC (*p* = 0.080). Moreover, in the 9M condition, a trend toward an increased Cmax for both insulin and GLP-1 was observed (*p* = 0.055 and *p* = 0.083, respectively), and the iAUC of GLP-1 was significantly increased after the intervention (*p* = 0.049). Glucagon, GIP, and the insulin sensitivity index did not differ significantly between the 9M and 3M conditions. In the analysis of temporal pattern, the linear mixed effects model revealed the lack of a primary effect of the intervention on fluctuations in blood glucose, insulin, and GLP-1 (*p* = 0.105, *p* = 0.099, and *p* = 0.493, respectively), the lack of an effect of time (*p* = 0.152, *p* = 0.193, and *p* = 0.521, respectively), and the lack of an intervention × time interaction (*p* = 0.289, *p* = 0.132, and *p* = 0.429, respectively). Blood glucose at 1 h after drinking the 75-g glucose solution was significantly lower in the 9M condition compared with the 3M condition (*p* = 0.020).

## 4. Discussion

The present study examined the influence of a meal frequency intervention over a 3-day period on glucose metabolism and secretion of gastrointestinal hormones in adult males with normal glucose tolerance or impaired fasting glucose using a CGMS and post-intervention OGTT. The 24-h mean blood glucose measured by the CGMS did not differ significantly between the 3M and 9M conditions. The daytime and nighttime maximum blood glucose and glucose range were significantly decreased, and the daytime minimum blood glucose was significantly increased in the 9M condition compared with the 3M condition. In the IFG group, significant reductions in blood glucose were detected by the OGTT in the 9M condition compared with the 3M condition. One potential explanation for the improvements observed in the 9M condition is the induction of GLP-1 secretion and improvement in insulin secretion.

Our findings are consistent with previous studies reporting reduced daily mean glucose concentrations and fluctuations when meal frequency is increased while keeping total caloric intake constant. For example, Jenkins et al. [17] compared a 13-meal/day diet (nibbling diet) and a 4-meal/day diet (3 meals + 1 snack) in a study targeting 11 patients with T2DM, and reported that the nibbling diet reduced blood glucose and blood insulin. According to a report by Munsters and Saris [22] comparing a 14-meal/day diet and a 3-meal/day diet in healthy individuals using a CGMS, those on the 14-meal/day diet exhibited a significantly reduced AUC of daytime blood glucose and stabilization of blood glucose fluctuations.

In the present study, the 24-h mean blood glucose as measured by the CGMS did not differ between the 3M and 9M conditions in subjects with either NGT or IFG. The daytime (07:00-23:00) maximum blood glucose and glucose range, as well as nighttime (23:00-07:00) maximum blood glucose, however, were significantly reduced in the 9M condition compared with the 3M condition, with a significantly smaller range of blood glucose fluctuations. In particular, in the IFG subjects, blood glucose levels after dinner were maintained at higher level in the 3M conditions, compared to the 9M conditions, and remained high levels of blood glucose during sleep. Previous study in the healthy individuals have found similar result [30]. Since blood glucose levels during sleep can be continuously measured by CGMS, further study is needed for new aspects. The strength of the present study is that we examined the effects of meal frequency during strictly management on meal timing and contents, activities and sleep-time for 3 days. Most of our subjects spent the intervention period in the outpatient unit under sedentary conditions, and thus their energy balance was uniform, allowing us to specifically assess the effect of altering the distribution of nutrient intake. In a study by Kanaley et al. [31], obese subjects with NGT on a 6-meal/day diet (1 meal every 2 h) and those on a 3-meal/day diet (1 meal every 4 h) exhibited fluctuations in the 1-day glucose concentration and insulin. In that study, while the AUC of glucose between the two meal frequencies did not differ, the AUC of insulin was significantly larger in the 3-meal/day group compared with the 6-meal/day group. Although we obtained continuous measurements of blood glucose during the intervention period, we did not measure insulin. It is possible, however, that a similar phenomenon occurred in our subjects. In subjects with NGT who can maintain insulin secretion despite reductions in meal frequency, insulin secretion is induced on a chronic basis to maintain a certain blood glucose concentration, suggesting that increased meal frequency is beneficial [32]. This possibility could be confirmed by studying subjects with varying degrees of glucose tolerance.

GLP-1 is a gastrointestinal hormone secreted by L cells in the lower small intestine starting from approximately 30 min after a meal in response to sugar and fat in the meal and bile acid produced during digestion [33,34]. GLP-1 is thought to play an important role in treating diabetes and is a target of many therapeutics given its ability to stimulate the secretion of insulin, but not glucagon, in the pancreas. A study comparing GLP-1 secretion between healthy people and patients with diabetes found that GLP-1 secretion was attenuated in the diabetic patients [35,36,37]. Furthermore, although the underlying mechanism of that relationship is unclear, many studies report that fasting and postprandial GLP-1 secretion is attenuated in obese individuals, independently of differences in glucose tolerance [38,39,40,41]. In the present study, while the mechanism remains unknown, GLP-1 secretion improved when fluctuations in glucose during the day were attenuated by increasing meal frequency while keeping the 3-day total caloric intake constant, particularly in subjects with IFG. Previous studies on the influence of diet (e.g., Western diet) and eating patterns on GLP-1 secretion reported a relationship between GLP-1 secretion and eating patterns [42,43]. Holiday and Blannin reported improvements in GLP-1 secretion, inhibition of ghrelin secretion, and reduced appetite by temporary exercise, although the underlying mechanism is unknown [44]. The mechanisms underlying the effects of eating patterns, energy balance, and intermittent hyperglycemia on GLP-1 secretion require further evaluation.

This study has several limitations. First, the number of subjects was relatively small. The necessary number of subjects could not be used for the power calculation due to schedule limitations. The clinical significance of a change in glucose metabolism must be further investigated. Second, our study population was limited to male subjects with NGT and IFG. Thus, it is unclear whether we would observe induced GLP-1 secretion and improved glucose metabolism in a different population (e.g., diabetic patients). Further larger and comprehensive studies in diabetic patients are needed for identifying the health relevance of these observations.

## 5. Conclusions

In conclusion, in adult males with NGT and those with IFG, the 3-day meal frequency intervention did not affect the 24-h mean blood glucose, but reduced the maximum blood glucose and glucose range in the 9M condition compared with the 3M condition. Moreover, in subjects with IFG, the OGTT in the 9M group revealed significantly reduced blood glucose compared with the 3M group. One possible reason for this is the induction of GLP-1 secretion and trend toward improved insulin secretion due to increased meal frequency.

## Figures and Tables

**Table 1:** Subject characteristics ^1^.

	NGT	IFG	*p* Values
Number (men)	9 (9)	9 (9)	
Age (years)	47 ± 2	51 ± 2	0.220
Weight (kg)	73.0 ± 4.7	76.1 ± 4.2	0.632
BMI (kg/m^2^)	25.4 ± 1.2	26.3 ± 1.5	0.616
BMR (kcal/d)	1530 ± 62	1556 ± 56	0.758
SBP (mmHg)	126.7 ± 4.5	135.2 ± 4.4	0.194
DBP (mmHg)	76.6 ± 3.9	85.7 ± 3.2	0.091
Glucose (mg/dL)	87 ± 2	114 ± 2	<0.001
Insulin (mU/mL)	5.8 ± 0.9	7.7 ± 1.2	0.215
HbA1c (%)	5.0 ± 0.1	5.9 ± 0.1	<0.001
HOMA-IR	1.2 ± 0.2	2.2 ± 0.3	0.027
Triglycerides (mg/dL)	186 ± 30	132 ± 14	0.123
HDL-cholesterol (mg/dL)	60 ± 4	47 ± 3	0.023
LDL-cholesterol (mg/dL)	138 ± 12	139 ± 10	0.977
FFA (mEq/L)	0.60 ± 0.03	0.71 ± 0.06	0.124

^1^ Data are expressed as mean ± SD. BMI, body mass index; BMR, basal metabolic rate; DBP, diastolic blood pressure; FFA, free fatty acid; HbA1c, hemoglobin A1c, HDL, high-density lipoprotein; HOMA-IR, homeostasis model assessment-insulin resistance; IFG, impaired fasting glucose; LDL, low-density lipoprotein; NGT, normal glucose tolerance; SBP, systolic blood pressure.

**Table 2:** 24-h glucose indices from CGMS ^1^.

	NGT			IFG		
	3M	9M	*p* Values	3M	9M	*p* Values
Mean (mg/dL)	112 ± 15	105 ± 15	0.132	139 ± 17	137 ± 10	0.800
SD ^2^ (mg/dL)	27 ± 10	19 ± 8	0.034	36 ± 9	24 ± 10	0.014
AUC (mg/dL×h)	2680 ± 361	2498 ± 352	0.132	3314 ± 396	3283 ± 250	0.804
Maximum (mg/dL)	184 ± 40	150 ± 20	0.016	233 ± 36	195 ± 30	0.016
Minimum (mg/dL)	69 ± 17	68 ± 13	0.923	75 ± 17	87 ± 14	0.185
Max-min range (mg/dL)	115 ± 42	82 ± 26	0.039	158 ± 31	108 ± 37	0.008
>180 mg/dL time (min)	73 ± 79	0 ± 0	0.025	193 ± 184	103 ± 140	0.172
<70 mg/dL time (min)	84 ± 122	51 ± 119	0.599	34 ± 60	0 ± 0	0.130

^1^ Data are expressed as mean ± SD. ^2^ SD was calculated using mean data from each subject (24-h SD). AUC, area under the curve; CGMS, continuous glucose monitoring system; IFG, impaired fasting glucose; NGT, normal glucose tolerance; 3M, three meals; 9M, nine meals.

**Table 3:** Fasting and postprandial glucose, insulin, insulin sensitivity indices, glucagon, GLP-1, and GIP during the OGTT ^1^.

	NGT			IFG		
	3M	9M	*p* Values	3M	9M	*p* Values
**Glucose**						
Fasting (mg/dL)	84 ± 4	82 ± 5	0.201	106 ± 13	105 ± 8	0.528
Cmax (mg/dL)	166 ± 40	160 ± 38	0.387	249 ± 52	236 ± 40	0.042
iAUC (mg/dL×h)	120 ± 67	112 ± 86	0.691	285 ± 123	242 ± 119	0.080
**Insulin**						
Fasting (μU/ml)	4.4 ± 1.2	4.6 ± 1.5	0.697	7.1 ± 3.5	6.7 ± 3.5	0.325
Cmax (μU/ml)	66.3 ± 38.4	66.1 ± 44.0	0.990	88.5 ± 76.7	118.9 ± 112.9	0.055
iAUC (μU/ml×h)	114.5 ± 63.6	117.0 ± 77.0	0.915	171.7 ± 140.5	213.6 ± 201.4	0.119
**Insulin sensitivity indices**						
HOMA-IR^2^	0.91 ± 0.27	0.93 ± 0.32	0.856	1.88 ± 1.04	1.70 ± 0.87	0.236
QUICKI	1.15 ± 0.13	1.16 ± 0.15	0.809	1.30 ± 0.21	1.28 ± 0.21	0.292
Matsuda index	9.06 ± 3.14	9.53 ± 4.20	0.641	5.16 ± 2.30	5.05 ± 2.03	0.789
Insulinogenic index	2.44 ± 5.51	1.05 ± 0.85	0.431	0.36 ± 0.23	0.42 ± 0.28	0.396
**Glucagon**						
Fasting (pg/mL)	2.39 ± 3.24	2.28 ± 3.35	0.605	2.16 ± 3.43	2.49 ± 3.49	0.666
Cmax (pg/mL)	3.11 ± 3.96	2.97 ± 3.65	0.347	3.08 ± 3.43	3.63 ± 3.80	0.602
iAUC (pg/dL×h)	0.49 ± 1.44	0.74 ± 3.42	0.829	−0.61 ± 3.64	0.85 ± 3.30	0.262
**GLP-1 (active)**						
Fasting (pmol/L)	0.4 ± 0.8	50.3 ± 21.8	0.345	59.2 ± 60.2	35.9 ± 21.1	0.364
Cmax (pmol/L)	7.5 ± 5.9	7.9 ± 4.6	0.826	7.7 ± 3.7	10.3 ± 5.6	0.083
iAUC (pmol/L×h)	10.4 ± 8.8	14.8 ± 17.9	0.437	10.0 ± 6.4	13.7 ± 6.0	0.049
**GIP (total)**						
Fasting (pg/mL)	62 ± 29	50 ± 22	0.345	59 ± 60	36 ± 21	0.364
Cmax (pg/mL)	478 ± 268	467 ± 239	0.750	453 ± 249	437 ± 260	0.733
iAUC (pg/mL×h)	855 ± 416	473 ± 158	0.774	1159 ± 772	1098 ± 657	0.644

^1^ Data are expressed as mean ± SD. ^2^ HOMA-IR and QUICKI were calculated from fasting plasma glucose and insulin during the OGTT. The insulinogenic index was calculated from fasting plasma glucose, insulin, and plasma glucose and insulin 60 min after starting the OGTT. The Matsuda index was calculated as described in a previous report [29]. iAUC, incremental area under the curve; CGMS, continuous glucose monitoring system; GIP, glucose-dependent insulinotropic polypeptide; GLP-1, glucagon-like peptide-1; IFG, impaired fasting glucose; NGT, normal glucose tolerance; 3M, three meals; 9M, nine meals.
